# The impact of logging roads on dung beetle assemblages in a tropical rainforest reserve

**DOI:** 10.1016/j.biocon.2016.11.011

**Published:** 2017-01

**Authors:** Felicity A. Edwards, Jessica Finan, Lucy K. Graham, Trond H. Larsen, David S. Wilcove, Wayne W. Hsu, V.K. Chey, Keith C. Hamer

**Affiliations:** a School of Biology, University of Leeds, Leeds LS2 9JT, UK; b Department of Animal and Plant Sciences, University of Sheffield, Sheffield S10 2TN, UK; c Science and Knowledge Division, Conservation International, 2011 Crystal Drive, Suite 500, Arlington, VA 22202, USA; d Woodrow Wilson School, Department of Ecology and Evolutionary Biology, Princeton University, Princeton, NJ 08544, USA; e Department of Ecology, Evolution, and Environmental Biology, Columbia University, New York 10027, USA; f Forest Research Centre, Sabah Forestry Department, P.O. Box 1407, 90715 Sandakan, Sabah, Malaysia

**Keywords:** Anthropocene era, Habitat loss, Infrastructure, Invertebrates, Scarabaeidae, Selective logging

## Abstract

The demand for timber products is facilitating the degradation and opening up of large areas of intact habitats rich in biodiversity. Logging creates an extensive network of access roads within the forest, yet these are commonly ignored or excluded when assessing impacts of logging on forest biodiversity. Here we determine the impact of these roads on the overall condition of selectively logged forests in Borneo, Southeast Asia. Focusing on dung beetles along > 40 km logging roads we determine: (i) the magnitude and extent of edge effects alongside logging roads; (ii) whether vegetation characteristics can explain patterns in dung beetle communities, and; (iii) how the inclusion of road edge forest impacts dung beetle assemblages within the overall logged landscape. We found that while vegetation structure was significantly affected up to 34 m from the road edge, impacts on dung beetle communities penetrated much further and were discernible up to 170 m into the forest interior. We found larger species and particularly tunnelling species responded more than other functional groups which were also influenced by micro-habitat variation. We provide important new insights into the long-term ecological impacts of tropical logging. We also support calls for improved logging road design both during and after timber extraction to conserve more effectively biodiversity in production forests, for instance, by considering the minimum volume of timber, per unit length of logging road needed to justify road construction. In particular, we suggest that governments and certification bodies need to highlight more clearly the biodiversity and environmental impacts of logging roads.

## Highlights

• Distinct edge effects along roads penetrate > 100 m into the logged forest interior.
• Vegetation changes however, differed within a maximum of 34 m of the road edge.
• Large diurnal, and tunnelling dung beetles are affected more by proximity to roads.
• Micro-habitat variations shown to influence large tunnelling species.

## 1 Introduction

Large areas of intact habitats rich in biodiversity are being opened up through extractive industries, including selective logging. Logging concessions account for ≈ 50% of the total area of tropical forests (Blaser et al., 2011), yet a largely overlooked impact of timber extraction is the creation of logging roads. Roads are an integral part of extractive industries, which require not only large transportation routes, but also secondary haulage trails and smaller access pathways, creating a sprawling ‘fishbone’ pattern of compressed barren surfaces mostly unpaved. For instance, in Borneo alone it is estimated there are over 270,000 km of such logging roads (Gaveau et al., 2014).

Roads can have negative ecological consequences by removing and degrading adjacent habitat, acting as barriers to dispersal, creating edge effects, and increasing the risk of road kill, fire, hunting and the colonisation by invasive species (Laurance et al., 2009, Benitez-Lopez et al., 2010, Rytwinski and Fahrig, 2013, Clements et al., 2014, Padmanaba and Sheil, 2014, Dar et al., 2015). The construction of roads across the tropics is therefore an urgent concern for conservation (Laurance and Balmford, 2013, Bicknell et al., 2015, Barber et al., 2014, Laurance et al., 2014), but further attention is needed to evaluate the long-term impacts of logging roads, which remain in the landscape long after logging has been completed (Gullison and Hardner, 1993, Ernst et al., 2016). Few studies, however have focused on the impacts of roads in tropical forests, let alone specific logging roads. Understorey bird communities were observed to decline, while termite community composition differed with proximity to unpaved road clearings in Amazonia (Laurance, 2004, Dambros et al., 2013). Dung beetle communities were negatively affected by logging dumps, skid trails and access roads shortly after logging in Malaysia (Hosaka et al., 2014a), and small mammal community composition differed between logging road types (variations in size, use and time since adandonment) in Central Africa (Malcolm and Ray, 2000). However, most studies of the impacts of logging on biodiversity have either explicitly or implicitly avoided roads in their sampling protocols, leading to calls for further studies of their impacts on biodiversity and ecosystem functioning (Hamer et al., 2003, Broadbent et al., 2008; Laufer et al., 2013).

This study is based within a 1 Mil ha logging concession in Sabah, Malaysian Borneo. Selective logging has been widespread in this region with extraction levels some of the highest globally (Cleary et al., 2007). In Sabah alone the total length of logging roads is estimated at > 37,000 km, with a density of 0.65 km per km^2^ (Gaveau et al., 2014). Timber extraction in the immediate area of our study site was completed 23 years ago, which provides an ideal opportunity to examine the long-term impacts of logging roads across a large scale and through continuous forest. We use dung beetles (*Coleoptera*: Scarabaeidae, Scarabaeinae) as our model taxon, as they are a key indicator group that contributes to diverse ecosystem processes (Gardner et al., 2008, Nichols et al., 2008) and is sensitive to environmental changes (Nichols et al., 2007).

The question of how far edge effects alongside roads penetrate into the forest is vital for understanding the overall impacts of logging on biodiversity. We address this key question by investigating the magnitude and extent of edge effects along logging roads (Harper et al., 2005, Harper and Macdonald, 2011), focusing on key vegetation and soil characteristics, and the species richness, community composition and abundance of different dung beetle functional groups. We then assess whether changes in vegetation characteristics can explain the observed changes in dung beetle community structure from the road edge to the logged forest interior. Finally, we compare logged forest with nearby primary forest to assess the additional impact of roads on dung beetle biodiversity, beyond that directly attributable to harvesting of timber.

## 2 Material and methods

### 2.1 Study location

The study site was the Yayasan Sabah (YS) logging concession in eastern Sabah (4° 58′ N, 117° 48′ E). Most of this concession (95%) has been selectively logged, including the 238,000 ha Ulu Segama-Malua Forest Reserve (US-MFR) of which 97,000 ha (41%) has undergone a single rotation of timber extraction (once-logged forest). Harvesting took place between 1987 and 1991, with a yield ≈ 115 m^3^ of timber per ha (Fisher et al., 2011), and 17% of the land area was marked by roads and skid trails (Pinard and Cropper, 2000). All roads used in this study are un-paved and are still in use and maintained, though not for logging activities. Vegetation along the road edge varies in height and complexity due to initial logging activities and more recent maintenance (e.g. repairing of collapsed bridges).

### 2.2 Dung beetle and vegetation sampling

**Fig. 1:**
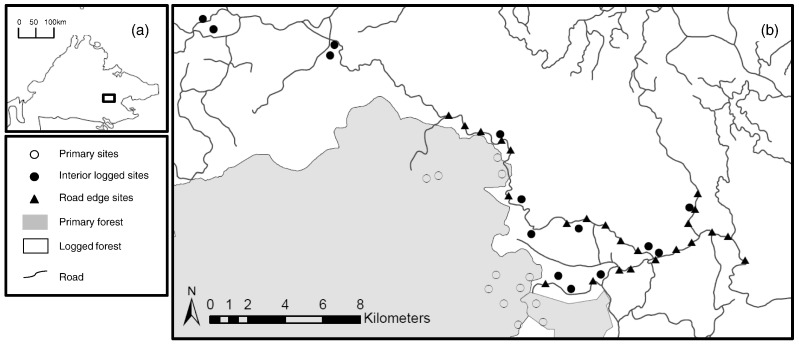
(a) A map of Sabah, Malaysian Borneo. The box outlines our study area. (b) A map of the study area. The grey solid area represents primary rainforest with the adjacent white area representing selectively logged rainforest. The symbols on the map identify sampling sites; open circles are within primary forest, solid black circles are within logged forest > 100 m from the road edge (interior logged forest), and solid black triangles are within logged forest up to 100 m from the road edge (road edge forest). In all cases, the mid-point of the sampling traps, at a given site, is represented on the map.

Fieldwork was conducted between August and October 2009, March and September 2011, and June and August 2014. To quantify changes in dung beetle assemblages in proximity to roads, we created 24 sampling plots which were widely spaced across the landscape with a minimum distance of 650 m (mean ± SD: 5.9 km ± 3.7) between plots. Each plot contained six traps at distances of 0 m, 6 m, 12 m, 25 m, 50 m and 100 m from the road edge (144 traps in total). To ensure independence of samples, traps were a minimum of 50 m apart (Larsen and Forsyth, 2005) in a staggered design following Barnes et al. (2014) (see Fig. A1). We considered that edge effects were unlikely to extend beyond 100 m (Benedick et al., 2006, Broadbent et al., 2008, Lucey and Hill, 2011, Gray et al., 2016) but to check whether or not this was the case and to determine how dung beetle assemblages differed between road edges and the interior of logged forest, we also placed traps (n = 58) 100 m apart along 14 transects at distances of 170 m to 550 m from the nearest road edge, with 4–5 traps per transect and a minimum distance of 500 m (mean = 11.9 km ± 8.5) between transects. We also sampled in primary forest, using 60 traps placed a minimum of 100 m apart along 12 transects of five traps each (mean distance between transects = 4.5 km ± 3.0)(see Fig. 1). We used standardised baited pitfall traps for all sampling. In each case a single trap, baited with human dung, was placed for four days and re-baited after 48 h, with beetles collected every 24 h (Edwards et al., 2011). We used reference collections (T. Larsen) housed at the Forest Research Centre, Sandakan, Malaysia and Smithsonian Museum, Washington DC, USA to assist identification.

Species vary greatly in their contributions to community biomass, which in turn can affect ecosystem functioning (Slade et al., 2007). To determine biomass per trap, we calculated the average mass (g) of each dung beetle species, multiplied this by the number of individuals in a trap, and summed across species. To determine body masses, individuals (up to a maximum of 15 per species) were dried for four days at 60 °C and weighed to the nearest 0.001 g using a precision balance (SBC 31; Scaltec Instruments GmbH, Germany). We also measured body length (base of head to tip of elytra) and width (distance between outer margins of elytra), to the nearest 0.1 mm using dial callipers and calculated body size (length ∗ width) to allow extrapolation of body mass for species that could not be weighed (Text B1, Fig. B1).

Additionally, 15 micro-habitat variables were measured at each sampling location within 100 m of the road edge (n = 144) and a subset of interior forest locations (n = 24) to determine how soil characteristics, leaf litter depth and vegetation structure, including tree characteristics, varied with distance from the road edge (Text C1).

### 2.3 Data analysis

#### 2.3.1 Edge effects

To examine how species richness, abundance and biomass of dung beetles, the abundance of different functional groups, vegetation structure and soil characteristics varied with distance from the road edge, we firstly used a piecewise regression to determine if a breakpoint (an abrupt change in a relationship) in our data was present. We ran a GLM with negative binomial error distribution (or in the case of certain vegetation variables a LM) with *distance* as a continuous variable, and then using this model we ran a piecewise regression (using the *segmented* package in R). To determine if the piecewise regression was the best model we compared AIC values (following Ochoa-Quintero et al., 2015, Magnago et al., 2015). The piecewise regression allowed us to determine whether there was a significant influence of distance and to identify any discrete breakpoint in a particular variable (P < 0.05).

Secondly, we assessed the magnitude of edge influence (MEI: Harper and Macdonald, 2011, Dodonov et al., 2013), described as the amount a particular variable differs at the ‘edge’ compared to the ‘interior’, using the calculation of $\mathrm{MEI}=\frac{\left(e-i\right)}{\left(e+i\right)}$ where *e* represents the average of a given variable at a *particular distance* from the edge, and *i* represents the average of a given variable within the *interior* habitat away from the edge. If a given distance from the edge (*e*) is equal to the interior (*i*) then MEI = 0, MEI is bounded by 1 and − 1 allowing for ease of comparison between variables. To examine the distance over which edge effects penetrated into the forest adjacent to roads (referred to as the distance of edge influence - DEI) we used a randomised method of edge influence (RTEI: Harper and Macdonald, 2011), described as the range of distances away from the edge (towards the interior) where there is a significant edge influence (Harper et al., 2005). This method follows three steps; i) observed MEI is calculated, ii) then randomised values of MEI are calculated from a complete variable pool (edge plus interior values) where the number of edge and interior sites are kept constant, and iii) then randomised values of MEI are compared to observed values to determine the significance of observed MEI (see Harper and Macdonald, 2011 for further details). The analyses were run separately for each distance (*e*) away from the road edge. This randomisation technique reduces type 1 errors by accounting for variation between sampling sites at a specific distance from the edge. We used 10,000 randomisations with a significance level of 0.05 for determining p-values. We also used this technique to assess the change in soil characteristics, leaf litter depth and vegetation structure away from the road edge.

Functional groups were determined using categories described by Slade et al. (2007), which represent the main behavioural guilds, diel activities and size categories of dung beetles, which have been found to relate to dung beetle functional activity within the study area (Slade et al., 2007, Slade et al., 2011).

#### 2.3.2 Community composition

To investigate how species composition changed with increasing distance from road edges, I used a non-metric multidimensional scaling ordination (Clarke and Warwick, 2001), using the Bray-Curtis dissimilarity measure (metaMDS function in *Vegan*; Oksanen et al., 2011). Communities were standardised as a proportion of the total number of individuals per distance class. To test for significant changes in community composition with distance from the road edge, I used a multivariate generalised linear model (GLM) framework, which allowed more accurate modeling of mean–variance relationships compared to pairwise matrix techniques (e.g. Bray–Curtis index), reducing type II errors (Warton et al., 2012). I used a negative binomial GLM, where multivariate p-values were calculated using PIT-trap bootstrapping with 1000 permutations, and were adjusted for multiple testing (anova.manyglm in Mvabund; Wang et al., 2014).

#### 2.3.3 Relationship of dung beetles to vegetation

To test whether there was a relationship between the changes in dung beetle community metrics and the observed vegetation changes, we ran generalised linear mixed effects models (GLMM) for each community metric (i.e. abundance, biomass) with a negative binomial error distribution. Each model included eight vegetation measures as predictors (successional vegetation, ground cover, canopy cover, the number of large and small trees, the height of large and small trees, and the girth of large trees) and ‘plot’ as a random factor to account for repeated measures. Those vegetation measures that showed no variation in the MEI analysis (see ‘edge effects’ above) were not included. We used a subset of the overall data where both vegetation and community data were available (n = 144 road edge plots with 24 plots per distance class plus 24 plots in the interior of logged forest, all surveyed in 2014). The vegetation variables were standardised to allow for analysis across different scales using the formula (*x* − mean(*x*)) / SD(*x*) where *x* is the vegetation variable to be standardised. To test whether or not our results were influenced by spatial autocorrelation we used a Monte-Carlo permutation test for Moran's I statistic (moran.mc function in *spdep*: Bivand and Piras, 2015), using our model residuals with 1000 repetitions. There was no evidence of spatial auto-correlation for any of the models (Moran's I: P ≥ 0.3).

#### 2.3.4 Spatial extent of logging roads and edges

To determine how far edge effects persisted away from the road edge, we examined at what distance, if any, there was a distinct change in each of our biodiversity metrics and micro-habitat variables. We then used a GIS layer of major and minor hard roads (excluding skid trails) across the YS logging concession to determine the area comprising logging roads and edges. This layer covers the majority of the YS concession and was the most detailed layer available to us. The area (km^2^) comprising logging roads and edges, beyond the linear feature of the road surface itself, was estimated as:

(1) $$\left(\text{Totalroadlength}\times \left[\text{limitofedgeeffects}\times 2\right]\right)$$

All statistical analyses were run in R v.3.1.1 (R Development Core Team, 2016 and all spatial analyses were run in ArcGIS 10.1 (ESRI, 2011).

## 3 Results

We sampled 23,570 individual dung beetles of 74 species. Those species recorded in the interior of logged forest were a subset of primary forest species, but we recorded an additional eight species at road edges, which were not found elsewhere in the study (or from previous studies in the same study area, Edwards et al., 2014) (Table D1).

### 3.1 Magnitude and extent of edge effects

Successional vegetation declined significantly with increasing distance from the road (piecewise regression: t = − 4.28, p < 0.001) while the number of small and large trees (t = 2.82, p = 0.005 and t = 2.78, p = 0.006 respectively), and the height and girth of large trees (t = 4.0, p < 0.001 and t = 2.86, p = 0.005 respectively) increased along the same gradient (Tables C1, C4, Fig. C2). A randomization test of edge influence (RTEI) supported these models but with the addition of canopy cover, ground cover and small tree height showing a significant decrease compared to interior logged forest (Table C4). There was, however, no effect of distance from edge on soil characteristics or leaf litter depth (Tables C1, C4, Fig. C1).

**Fig. 2:**
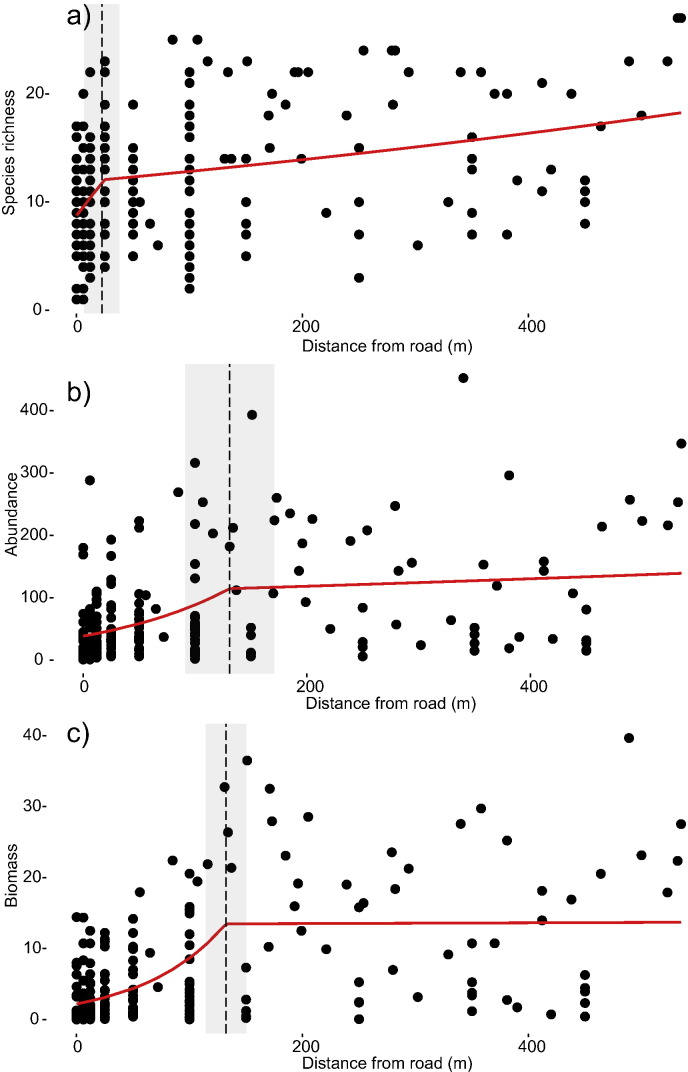
The effect of distance from the road edge (m) on the a) abundance, b) species richness, and c) biomass of dung beetles communities. Solid red lines are based on piecewise regression, dashed vertical lines represent significant breakpoints (P < 0.05), and grey shaded areas represent SE around breakpoint distance.

Abundance and biomass of dung beetles per trap both increased with increasing distance from the road (t = 3.73, P < 0.001 and t = 4.26, P < 0.001, respectively), with the greatest increase occurring around 130 m (Fig. 2). RTEI confirmed that the magnitude of the difference in each of these two response variables was significant up to 100 m from the road edge (Table C3, Fig. C3).

**Fig. 3:**
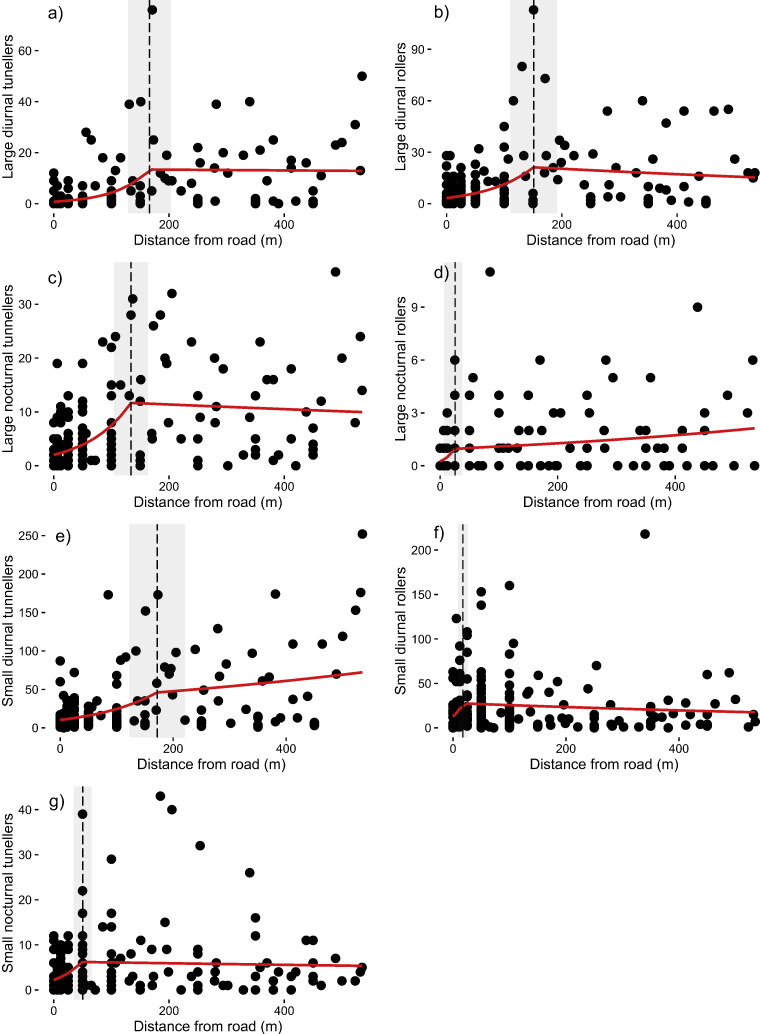
The effect of distance from the road edge (m) on the abundance of seven key dung beetles functional groups; a) large diurnal tunneller, b) large diurnal roller, c) large nocturnal tunneller, d) large nocturnal roller, e) small diurnal tunneller, f) small diurnal roller, g) small nocturnal tunneller. Solid red lines are based on piecewise regression, dashed vertical lines represent significant breakpoints (P < 0.05), and grey shaded areas represent SE around breakpoint distance.

**Fig. 4:**
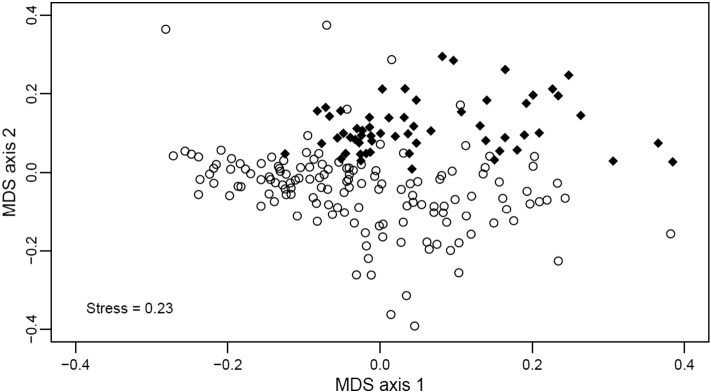
The relationship between non-metric multidimensional scaling (NMDS) ordination axis 1 and axis 2. Open circles represent traps between 0 and 100 m from the road edge, and solid diamonds represent traps in the interior logged forest, > 170 m from the road edge.

In terms of functional groups, large nocturnal tunnellers, large diurnal rollers and both large and small diurnal tunnellers all increased significantly in abundance with increasing distance from the road edge (t = 5.32, P < 0.001, t = 4.86, P < 0.001, t = 4.82, P < 0.001, and t = 4.30, p < 0.001 respectively) up to a distance 130 m (Fig. 3). Large nocturnal rollers and small nocturnal tunnellers (t = 2.78, p = 0.006 and t = 3.10, p < 0.002, respectively) changed in abundance much closer to the road edge (< 50 m, Fig.3d, g), whereas small diurnal rollers were unaffected (P = 0.19, Fig. 3f). The magnitude of edge effects and the RTEI confirmed similar patterns, highlighting significantly different abundances up to 100 m from the road edge compared to interior logged forest for the majority of functional groups, with the exception of small nocturnal tunnellers (DEI of 25 m) and small diurnal rollers which were found not to differ (Table C3, Fig. C3). Finally, community composition indicated that beetle assemblages within 100 m of the road were significantly different from those at greater distances (Fig. 4; manyglm: Wald statistic = 26.25, P = 0.001). There was no evidence of spatial auto-correlation for any of the above models (Moran's I: P ≥ 0.1).

### 3.2 Relationship of dung beetles to vegetation

The overall biomass of dung beetles and the abundance of large diurnal and nocturnal tunnellers were all significantly positively related to ground cover, while the abundance of large and small nocturnal tunnellers were significantly negatively related to the density of early successional vegetation (Table C5). Some additional variables also increased significantly with increasing densities or sizes of tress but there was no relationship between the abundance or biomass of rollers and any of the measured vegetation characteristics (Table C5).

### 3.3 Spatial extent of logging roads and edges

We estimated that the area affected by logging roads within the YS concession (i.e. including edge effects) was 817 km^2^, which is 9.0% of the total area of logged forest within the concession. Accounting for this area of road edge forest resulted in an additional decline of 3–8% in overall community metrics and in the abundance of different functional groups in the logged landscape compared to the effect of timber removal only (Table 1).

## 4 Discussion

### 4.1 Edge effects

This study provides one of the first examples of how tropical biodiversity responds to logging roads per se (Laurance, 2004, Hosaka et al., 2014a), and also assesses what the broader impact of timber extraction is by accounting for the hidden additional effects of logging roads. These results show clear evidence that while vegetation structure and composition were significantly affected up to 34 m from the road edge, impacts on dung beetle communities penetrated much further and were discernible up to 170 m into the forest interior (Fig. 2, Fig. 3, C3b). Moreover, these changes were observed > 20 years after timber extraction ended, supporting previous findings of the long term impacts of roads in Central Africa and Amazonia (Malcolm and Ray, 2000, Laurance et al., 2004) and highlighting a need for longstanding conservation efforts.

These results for dung beetles accord with the median extent of edge effects within forest fragments in the Brazilian Amazon (100 m; Broadbent et al., 2008), suggesting that transection of forest by logging roads could be considered akin to fragmentation in terms of edge effects. We also found that the distances of edge influence extended much further than previously recorded in Southeast Asian forests that had been selectively logged < 18 months previously (< 10 m; Hosaka et al., 2014b), possibly indicating a time lag in species' responses. More broadly, the declines we recorded in dung beetle community and functional metrics between road edges and elsewhere within logged forest exceeded the difference between primary forest and the interior of logged forest, highlighting the stark decline in biodiversity in proximity to roads (Table 1).

### 4.2 Impact of roads on logged forest biodiversity

The changes we recorded in community composition indicate potential changes in the ecosystem functions provided by dung beetles within logged forest. The magnitude of edge influence was greater for diurnal tunnellers and larger species compared to rollers and smaller species (Fig. 2, Table C3, Fig. C3). Notably the decline of large tunnellers, which have been shown to remove more dung than the other functional groups (Slade et al., 2007), could have important implications for the overall rate of dung removal. Furthermore the decline in larger species may contribute to changes in local-scale species interactions including the greater numerical dominance of smaller species, particularly diurnal rollers, in road edge forest (Table 1). Tunnelling species, including larger species, were shown to associate with greater tree density and structure but with ground cover present. Micro-habitat and micro-climatic changes, have been highlighted as a key determinant in changes in small mammal and dung beetle populations, specifically a loss of canopy cover, following road creation (Malcolm and Ray, 2000, Hosaka et al., 2014b) but also in other extreme environments (oil palm plantations and logging yards) which represent similar extreme changes in habitat structure as with roads and logged forest edge (Edwards et al., 2014, Hosaka et al., 2014a). These findings highlight the unknown interactions between functional traits and community assembly, and the need for a greater understanding of assembly filters in varied disturbed habitats (Pollock et al., 2012, Van der Plas, 2012).

### 4.3 Management implications

Roads are an essential but financially costly element of logging activities (Putz et al., 2008, Medjibe and Putz, 2012), and this study highlights the long-lasting ecological consequences of road creation during selective logging, above and beyond the direct effects of the removal of trees. Consequently, there are incentives and benefits to both concession holders and biodiversity conservation to improve the design and implementation of logging roads.

The logging concession we studied is a relatively closed area with tightly controlled access, low traffic volumes and minimal human settlements (principally three well-contained forest research stations and a tourist lodge), and thus further degradation has been minimal while promoting forest recovery. Our results show that even under this ‘best-case scenario’ there are significant impacts of logging roads. Furthermore, where logging roads facilitate uncontrolled access to the forest long after logging has ceased, edge effects could be greatly exacerbated and penetrate further into the logged forest interior. Thus we support suggestions for the closure (permanent or temporarily) of logging roads, where appropriate, once timber extraction has been completed, to facilitate forest recovery and discourage encroachment (Bicknell et al., 2015, Kleinschroth et al., 2016).

In conclusion we suggest that governments and certification bodies (e.g. the Forest Stewardship Council - FSC) need to highlight more clearly the biodiversity and environmental impacts of logging roads. We also encourage the increased use of reduced impact logging techniques (RIL; Edwards et al., 2012, Putz et al., 2012, Bicknell et al., 2014) and suggest that the planning of roads within logging concessions needs to take further steps to preserve forest, for instance by considering the minimum volume of timber that would need to be extracted per unit length of logging road in order to justify road construction. This is a timely and important discussion as large logging concessions open up across South-east Asia, South America and tropical Africa, and there is a need and desire to encourage more sustainable and conservation-focused planning for logging activities.

## Figures and Tables

**Table 1:** Biodiversity metrics (mean [SE]) for dung beetles sampled within primary forest, > 100 m from the nearest road within logged forest (interior), within 100 m of logging roads (road edge) and the combined logged landscape⁎ in Sabah, Malaysian Borneo.

Metric	Primary forest	Logged forest		
		Interior	Road edge	Combined
Overall community				
Species richness	18.5 (0.7)	15.8 (0.8)	10.7 (0.4)	13.9 (0.4)
Abundance	147.7 (14.3)	129.3 (14.0)	48.6 (4.5)	122.0 (7.4)
Biomass (g)	24.7 (2.3)	14.1 (1.4)	3.4 (0.3)	14.7 (0.8)
Abundance of functional groups				
Large diurnal tunnellers	9.6 (1.1)	13.0 (1.9)	0.9 (0.2)	6.2 (1.0)
Large nocturnal tunnellers	8.5 (1.0)	11.3 (1.2)	3.1 (0.3)	11.9 (0.7)
Small diurnal tunnellers	43.5 (5.0)	55.6 (7.4)	12.7 (1.3)	51.7 (3.8)
Small nocturnal tunnellers	17.5 (1.6)	6.4 (1.2)	4.4 (1.0)	10.6 (0.8)
Large diurnal rollers	26.8 (3.3)	20.4 (3.1)	4.3 (0.6)	19.0 (1.6)
Large nocturnal rollers	5.4 (0.9)	1.5 (0.3)	0.6 (0.1)	1.4 (0.2)
Small diurnal rollers	33.7 (5.8)	21.0 (4.3)	22.6 (2.5)	21.1 (2.3)

⁎ Means for combined logged forest are weighted by the proportions of interior and road edge forest within the logged landscape of the study concession area.

## Supplementary data

Supplementary material Image 1
