# Supplementary material for: The impact of logging roads on dung beetle assemblages in a tropical rainforest reserve
Source: Biol Conserv. 2017 Jan;205:85–92. doi: 10.1016/j.biocon.2016.11.011 (PMC5239768; doi:10.1016/j.biocon.2016.11.011)
Supplement: Supplementary file 1 — Supplementary material [file mmc1.docx]

**Supplementary Material**

**The impact of logging roads on dung beetle assemblages in a tropical rainforest reserve**

**Felicity A. Edwards^1*^ Jessica Finan^1^, Lucy K. Graham^2^, Trond H. Larsen^3^, David S. Wilcove^4^, Wayne W. Hsu^5^, V. K. Chey^6^ and Keith C. Hamer^1^**

^1^ *School of Biology, University of Leeds, Leeds, LS2 9JT, UK.*

*^2^ Department of Animal and Plant Sciences, University of Sheffield, Sheffield, S10 2TN, UK.*

*^3^ Science and Knowledge Division, Conservation International, 2011 Crystal Drive, Suite 500, Arlington, Virginia 22202 USA*

*^4^Woodrow Wilson School and Department of Ecology and Evolutionary Biology, Princeton University, Princeton, NJ 08544, USA*

*^5^ Department of Ecology, Evolution, and Environmental Biology, Columbia University, New York 10027 USA*

*^6^ Forest Research Centre, Sabah Forestry Department, P.O. Box 1407, 90715,* *Sandakan, Sabah, Malaysia*

Contents:

Appendix A

Fig. A1: Placement of traps within plots measuring edge effects

Appendix B

Text B1: Description of biomass extrapolation

Fig. B1: Biomass regression calculation

Appendix C

Text C1: Description of micro-habitat variables collected

Table C1: Summary of piecewise regression outputs for vegetation structure and soil characteristics

Table C2: Summary of RTEI output for vegetation variables

Table C3: Summary of RTEI output for community and functional metrics

Table C4: Summary of mean micro-habitat variable per distance category

Table C5: GLMM model outputs investigating the relationship between community metrics and the abundance of functional groups with key micro-habitat variables

Figure C1: The variation in the magnitude and the extent of edge influence for vegetation structure and soil characteristics

Figure C2: The variation in the magnitude and the extent of edge influence for tree characteristics

Figure C3: The variation in the magnitude and the extent of edge influence for community and functional metrics

Appendix D

Table D1: Summary of mean number of species per trap

**Appendix A**

Figure A1: The placement of traps (brown circles) within plots measuring edge effects. Traps were at least 50m apart and at distances of; 0m, 6m, 12m, 25m, 50m and 100m from the road edge (grey dashed line). Inset picture shows a section of a logging road in the study are

**Appendix B**

Text B1: Biomass was calculated using a regression of body size (mm) against dry mass (g) based on 23 dung beetle species of 181 individuals (regression adj-R^2^ = 0.96, Fig. B1), excluding the three largest species because these skewed the data for smaller species. The biomass of these larger species was calculated separately.

Figure B1: The relationship between body size (mm) and dry mass (g) based on 23 dung beetle species (181 individuals) from Sabah, Malaysian Borneo, used to extrapolate dry mass for the remaining species.

**Appendix C**

Text C1: To determine how the soil characteristics, leaf litter depth and vegetation structure varied with distance from the road edge, 15 micro-habitat variables were measured which are likely to be important for dung beetles. *Soil moisture* (wet mass-dry mass/dry mass)*100) and *bulk density (*dry soil weight (g)/soil volume (cm^3^)) were measured as the mean from three soil cores randomly taken within 0.5m of the trap at a depth of 0-10cm. The wet weight, measure to the nearest 0.1g, and length of soil cores was taken before cores were dried for four days in an oven at 60°C, before being weighed again. *Soil temperature* was measured using a temperature probe (VWR digital thermometer) next to the trap at a depth of approximately 10cm. L*eaf litter depth* was measured at ten random points, five within 1m and five within 2m of the trap and the average taken. The *percentage ground cover* was estimated within 2m^2^ around the trap and included all vegetation up to 0.5m above the ground. *Canopy openness* was measured using a spherical densitometer above the trap. The vegetation stand was measured by the *girth* at breast height (~1.2 m) and estimated *height* of the eight nearest small (≤60cm GBH) and large (>60cm GBH) trees to the trap. *Small tree density* was measured as the number of small trees within 10m^2^ of the trap and *large tree density* was measured as the number of large trees within 30m^2^ of the trap. *Vine density* was estimated for large (> 5cm diameter) and small (≤ 5cm diameter) vines, and *successional vegetation density* was estimated for bamboo, climbers, grass, ginger, ferns and rattan. Both vine density and successional vegetation were estimated using a categorical scale; 0 – none present, 1 – one or two plants/clumps, 2 – less than 25% cover, 3 – 26-50% cover, 4 – 51-75% cover, 5 – 76-100% cover, and the sum taken per trap. To determine how the vegetation structure altered along the gradient of road edge to interior logged forest we used linear models for each of our 15 environmental variables.

**Table C1:** Summary of piecewise regression outputs for the impact of distance away from the road edge and vegetation structure and soil characteristics. * indicates where linear models were shown (via AIC selection) to be the better model than a piecewise regression.

**Table C2**: The variation in the magnitude of the edge influence (MEI) for vegetation variables in road edge plots compared to interior logged forest. Distance is measured in metres away from the road edge. The MEI is bounded by 1 and -1, a positive value indicates a value at the edge>interior, a negative value indicates a value at the edge<interior, and a value of zero equals no difference between edge and interior forest. The p-values determine the distance of edge influence (DEI), P <0.05 indicates a significant difference at a given distance from the interior community. Significant results are highlighted in **bold**. Abbreviations refer to; ‘int’ = interior.

**Table C3**: The variation in the magnitude of the edge influence (MEI) for dung beetle community and functional metrics in road edge plots compared to interior logged forest. Distance is measured in metres away from the road edge. The MEI is bounded by 1 and -1, a positive value indicates a value at the edge>interior, a negative value indicates a value at the edge<interior, and a value of zero equals no difference between edge and interior forest. The p-values determine the distance of edge influence (DEI), P <0.05 indicates a significant difference at a given distance from the interior community. Significant results are highlighted in **bold**. Abbreviations refer to; *ldr*-large diurnal roller, *lnr*-large nocturnal roller, *sdr*-small diurnal roller, *ldt*-large diurnal tunneller, *lnt*-large nocturnal tunneller, *sdt*-small diurnal tunneller, *snt*-small nocturnal tunneller, and ‘int’ = interior.

**Table C4:** Mean (SD) micro-habitat variables in each distance category from the road edge and interior logged forest.


**Table C5:** GLMM model outputs investigating the relationship between community metrics and the abundance of functional groups with key micro-habitat variables. Abbreviations refer to SNR – small nocturnal roller, LDR – large diurnal roller, LNR – large nocturnal roller, SDT – small diurnal tunneller, SNT – small nocturnal tunneller, LDT – large diurnal tunneller, LNT – large nocturnal tuneller. Variables with a significance at P>0.05 are highlighted in **bold**.

**Figure C1:**


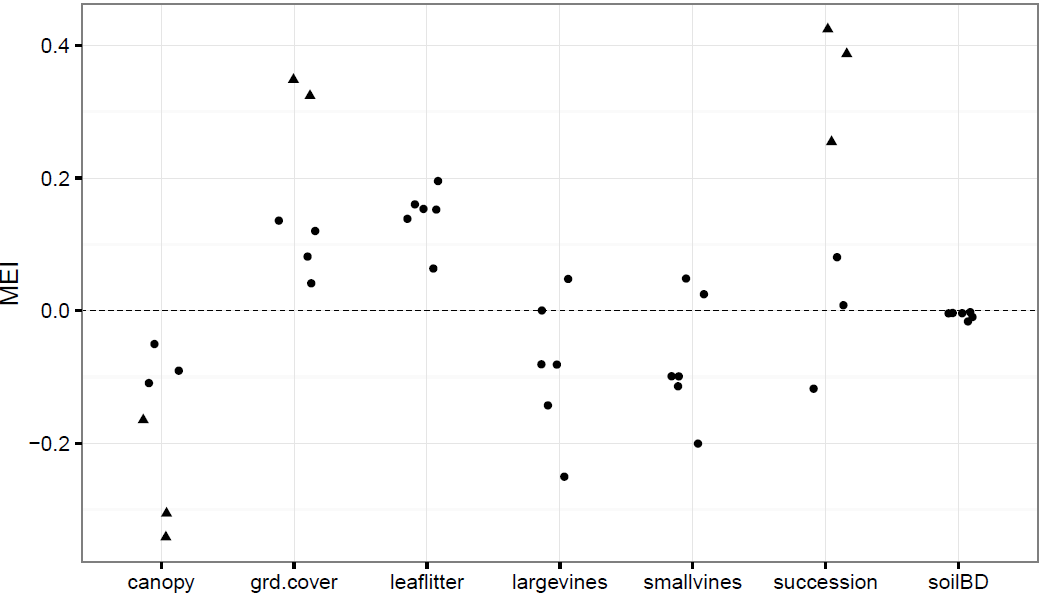


a)


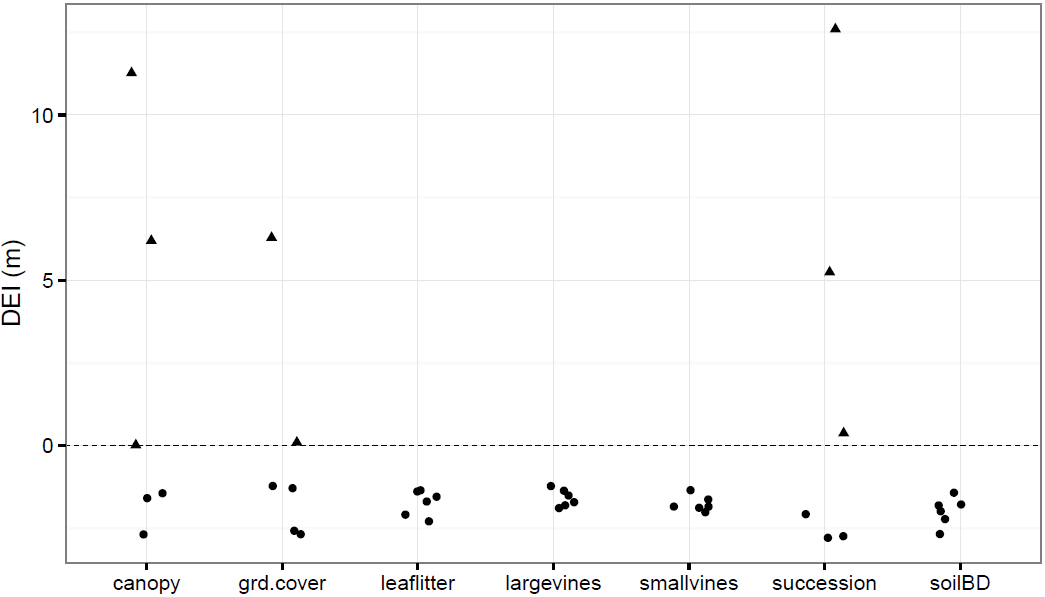


b)

**Figure C1:** The variation in the magnitude (a) and the extent (b) of edge influence for vegetation structure and soil characteristics. Triangles represent significant edge influence, while circles represent non-significance. The magnitude of the edge influence (MEI) is bounded by 1 and -1, a positive value indicates a value at the edge>interior, a negative value indicates a value at the edge<interior, and a value of zero equals no difference between edge and interior forest. The distance of edge influence (DEI) is measured in metres away from the road edge. X-axis labels refer to (unless clearly stated); *canopy*-canopy cover, *grd.cover*-ground cover, *succession*=successional vegetation, and *soilBD*-soil bulk density.

**Figure C2:**


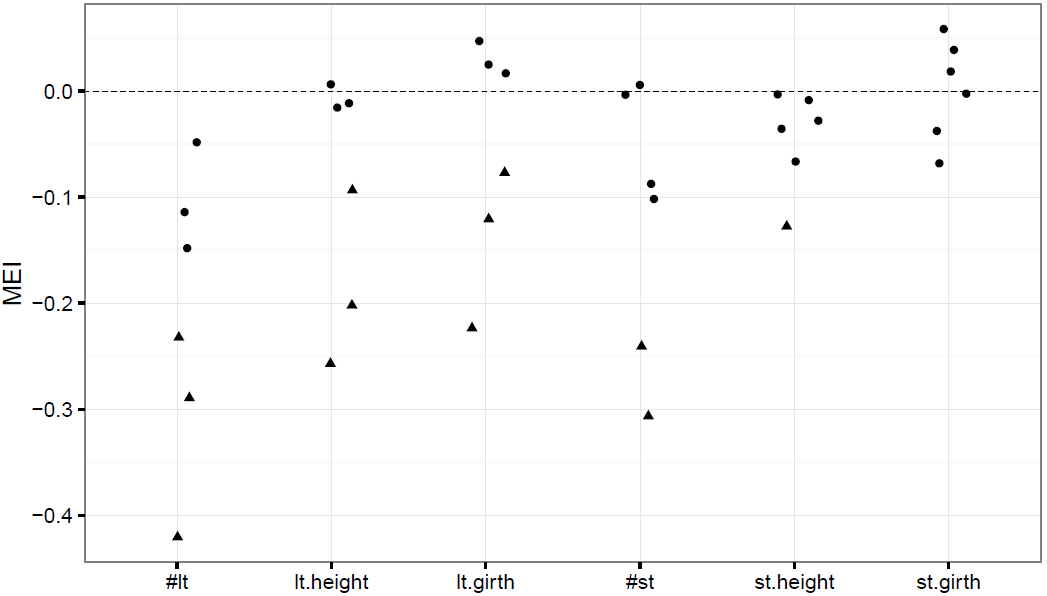


a)


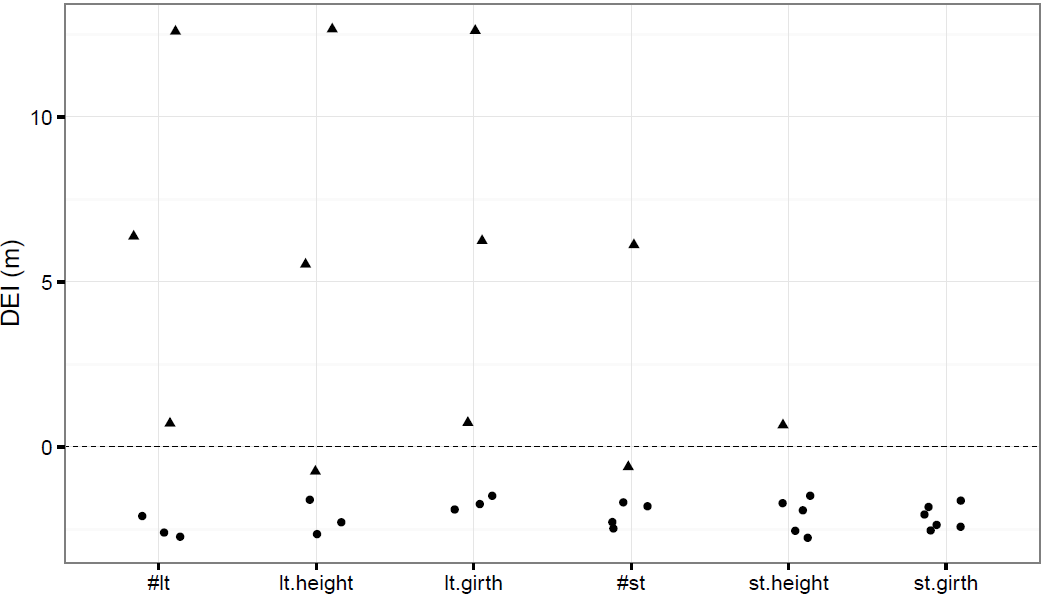


b)

**Figure C2:** The variation in the magnitude (a) and the extent (b) of edge influence for tree characteristics. Triangles represent significant edge influence, while circles represent non-significance. The magnitude of the edge influence (MEI) is bounded by 1 and -1, a positive value indicates a value at the edge>interior, a negative value indicates a value at the edge<interior, and a value of zero equals no difference between edge and interior forest. The distance of edge influence (DEI) is measured in metres away from the road edge. X-axis labels refer to (unless clearly stated); *#lt*-number of large trees, *lt.height*-large tree height, *lt.girth*=large tree girth, *#st*-number of small trees, *st.height*-small tree height, and *st.girth*=small tree girth.

**Figure C3:**


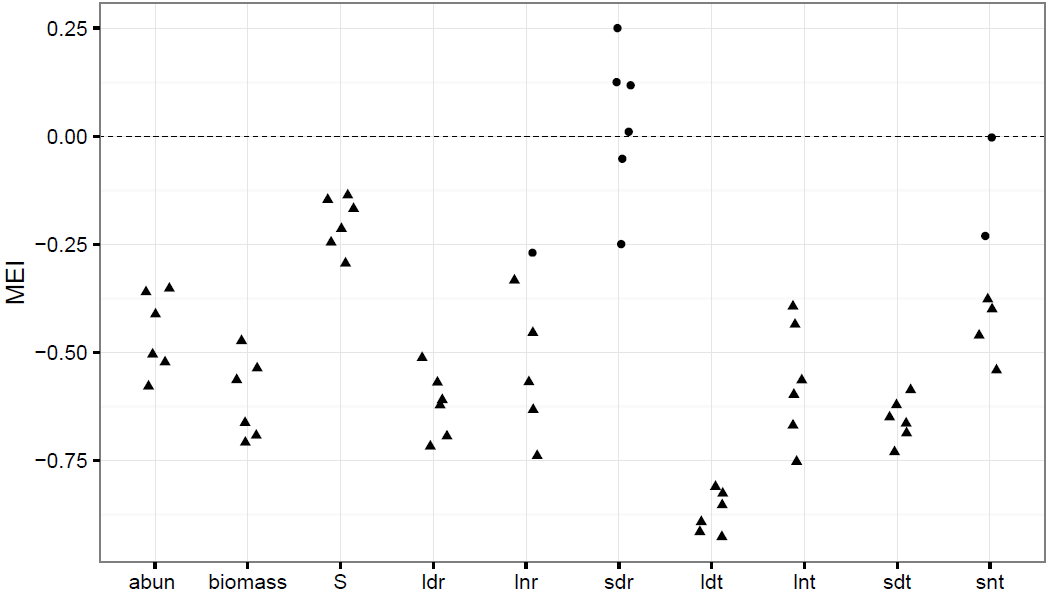


a)


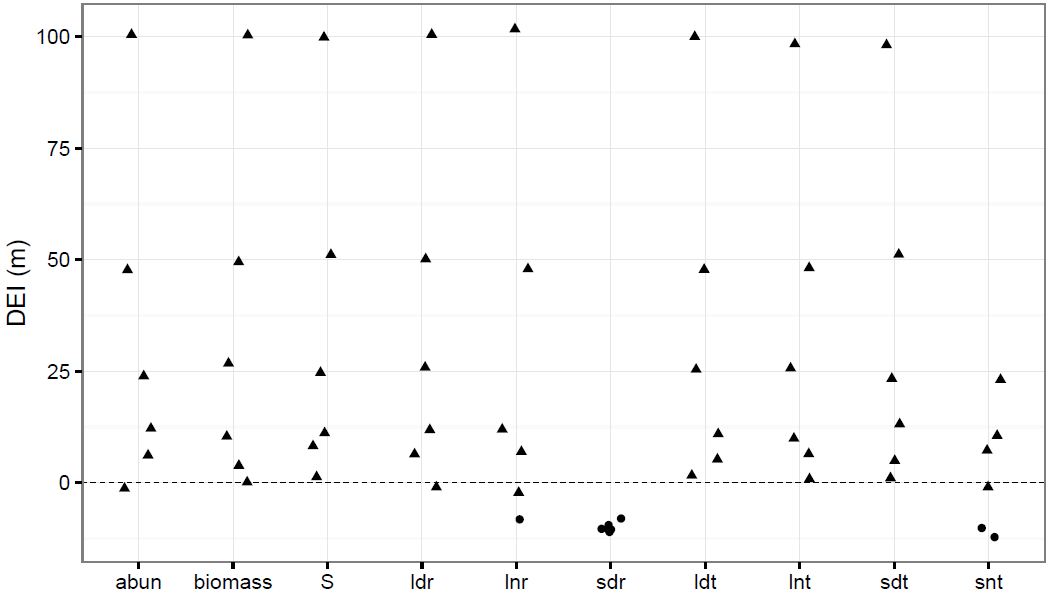


b)

**Figure C3:** The variation in the magnitude (a) and the extent (b) of edge influence for dung beetle community and functional metrics. Triangles represent significant edge influence, while circles represent non-significance. The magnitude of the edge influence (MEI) is bounded by 1 and -1, a positive value indicates a value at the edge>interior, a negative value indicates a value at the edge<interior, and a value of zero equals no difference between edge and interior forest. The distance of edge influence (DEI) is measured in metres away from the road edge. X-axis labels refer to (unless clearly stated); *abun*-abundance, *S*-species richness, *ldr*-large diurnal roller, *lnr*-large nocturnal roller, *sdr*-small diurnal roller, *ldt*-large diurnal tunneller, *lnt*-large nocturnal tunneller, *sdt*-small diurnal tunneller, and *snt*-small nocturnal tunneller.

**Appendix D**

Table D1: The mean number of individuals per trap across all species for each road edge distance category, interior logged forest and primary forest.
